# *Lactobacillus paracasei* LP18 ameliorated inflammation and intestinal barrier dysfunction in severe acute pancreatitis via gut microbiota-mediated regulation of butyrate metabolism

**DOI:** 10.3389/fmicb.2026.1765127

**Published:** 2026-02-12

**Authors:** Jianliang Cao, Anran Song, Qiang Zhang, Tangjuan Zhang, Xinya Jia, Bo Li, Chao Lan, Yuepeng Hu

**Affiliations:** 1 Department of Emergency Medicine, The First Affiliated Hospital of Zhengzhou University, Zhengzhou, China; 2 Henan Engineering Research Center for Cardiopulmonary and Cerebral Resuscitation, Zhengzhou, China; 3 Department of Physical Diagnosis, The First Affiliated Hospital of Zhengzhou University, Zhengzhou, China; 4 Translational Medicine Centre, The First Affiliated Hospital of Zhengzhou University, Zhengzhou, China

**Keywords:** butyrate metabolism, gut microbiota, intestinal barrier dysfunction, *Lactobacillus paracasei* LP18, severe acute pancreatitis

## Abstract

**Introduction:**

Severe acute pancreatitis (SAP) is frequently complicated by intestinal barrier disruption, bacterial translocation, and systemic inflammation. Emerging evidence indicates that gut microbial metabolic disturbances, particularly altered short-chain fatty acids (SCFAs), may contribute to epithelial injury, yet the mechanistic links between microbial metabolites and host inflammatory signaling remain insufficiently defined. We therefore investigated whether *Lactobacillus paracasei* LP18 protects against SAP-associated intestinal barrier dysfunction by reshaping gut metabolism, restoring butyrate availability, and attenuating NF-κB activation.

**Methods:**

A mouse model of SAP was established and animals received LP18 intervention. Intestinal injury and barrier integrity were evaluated by histopathology, tight junction protein assessment, and inflammatory cytokine measurements. Metabolic alterations were profiled using untargeted metabolomics and targeted quantification of SCFAs was performed. NF-κB signaling was assessed by measuring p65 phosphorylation and nuclear translocation. Correlation analyses were conducted to relate SCFA levels to barrier markers.

**Results:**

SAP induced pronounced epithelial injury, including villus atrophy, Tight junction disruption, elevated pro-inflammatory cytokines, and robust NF-κB activation. Untargeted metabolomics revealed extensive metabolic perturbations, with significant changes in butanoate metabolism and lipid-associated pathways. LP18 administration partially reversed these abnormalities and shifted the metabolomic profile toward a mucosa-protective signature. Targeted SCFA analysis showed marked butyrate depletion in SAP mice, whereas LP18 significantly increased butyrate levels and partially normalized acetate and propionate. Higher butyrate concentrations correlated with improved intestinal integrity and reduced inflammatory response. Mechanistically, LP18 suppressed inflammatory signaling by inhibiting p65 phosphorylation and nuclear translocation, consistent with attenuated NF-κB pathway activation.

**Conclusion:**

*Lactobacillus paracasei* LP18 attenuates SAP-associated intestinal inflammation and barrier dysfunction, primarily through modulation of gut microbial composition, restoration of butyrate-associated metabolic profiles, and suppression of NF-κB-related inflammatory signaling.

## 1 Introduction

Severe acute pancreatitis (SAP) is a life-threatening inflammatory condition marked by widespread acinar cell damage, systemic inflammatory response syndrome (SIRS), and a significant risk of multiorgan failure (Chen X. et al., 2024; Lee et al., 2024). Among the secondary complications of SAP, intestinal barrier failure serves a pivotal and amplifying function in the advancement of the disease (Agarwal et al., 2023; Zeng et al., 2024). The intestinal epithelium functions not only as a physical barrier but also as an immune-metabolic interface that modulates microbial interactions, nutrient uptake, and immune signaling (Haber et al., 2017; Allaire et al., 2018; Fedi et al., 2021). During SAP, compromised epithelial integrity results in increased intestinal permeability, bacterial translocation, and endotoxin influx into the systemic and portal circulation, thereby exacerbating pancreatic injury and contributing to hepatic inflammation and multi-organ failure (Tian et al., 2013; Wang et al., 2024). Therefore, delineating the mechanisms responsible for SAP-associated disruption of the intestinal barrier is essential for the development of innovative therapeutic approaches.

Emerging evidence increasingly implicates gut microbiota dysbiosis as a critical determinant in the pathophysiology of SAP (Wang et al., 2024). Clinical and experimental investigations have consistently demonstrated that severe acute pancreatitis (SAP) precipitates a rapid reduction in microbial diversity, depletion of beneficial commensal organisms, and proliferation of opportunistic pathogens (Glaubitz et al., 2023; Li X. et al., 2024). These modifications significantly disrupt microbial functional outputs, encompassing short-chain fatty acid (SCFA) production, bile acid metabolism, and amino acid fermentation, all of which are critical for maintaining intestinal immune homeostasis (Ye et al., 2021; Nista et al., 2025). Of these, butyrate, a prevalent SCFA generated through the microbial fermentation of dietary fibers, is of particular significance (Wang et al., 2022b, 2023). Butyrate functions as the principal energy substrate for colonocytes, promotes mucin synthesis, strengthens tight junction formation, and demonstrates significant anti-inflammatory and antioxidative properties (Salvi and Cowles, 2021; Foley et al., 2022). Decreased concentrations of butyrate have been associated with a diverse array of inflammatory conditions, encompassing inflammatory bowel disease, sepsis, and pancreatitis-associated enteropathy (Peña-Rodríguez et al., 2022). The composition of the gut microbiota significantly impacts butyrate synthesis. Key butyrate-producing genera such as *Faecalibacterium, Roseburia*, and *Eubacterium* are frequently markedly reduced in SAP (Fagundes et al., 2021; Peña-Rodríguez et al., 2022). Conversely, certain bacteria may disrupt butyrate metabolism by competing for substrates, generating inhibitory metabolites, or modifying microbial ecological networks (Liu et al., 2020; Gasaly et al., 2021). Emerging evidence indicates that not all *Lactobacillus* strains provide beneficial effects; instead, their influence is specific to the strain and dependent on the context (Eloe-Fadrosh et al., 2015; Chang et al., 2021; Roupar et al., 2023).

Although *Lactobacillus* species are conventionally considered probiotics with immunomodulatory and epithelial-supportive roles, recent research suggests that these effects are markedly strain-specific and context-dependent (Kim et al., 2025; Sun et al., 2025). Certain strains may be ineffective in providing benefits or may potentially worsen pathology in conditions of severe inflammation or microbial imbalance. LP18 was isolated from ‘Jiangshui', a traditional spontaneously fermented vegetable-based food widely consumed in Northwest China. Jiangshui fermentation relies on naturally occurring lactic acid bacteria that adapt to acidic and oxidative fermentation environments, making it a rich and ecologically relevant source for screening probiotic strains with enhanced antioxidant capacity and stress tolerance.Experimental evidence demonstrates that this strain significantly decreases serum uric acid levels in both hyperuricemic mouse models and human clinical trials, primarily by modulating the gastrointestinal microbiota. Given the significant changes in microbial ecology observed during SAP, it is conceivable that particular strains, such as LP18, could modify ecological networks or metabolic pathways in adverse manners, especially those associated with SCFA metabolism. Butyrate metabolism is subject to precise regulation by the gut microbial community. Classical butyrate producers are frequently diminished in SAP (Xia et al., 2023; Gates et al., 2024; Zhen et al., 2025). Simultaneously, the proliferation of specific non-butyrate-producing or metabolically competitive strains may further impair substrate availability and disrupt metabolic flux (Markandey et al., 2021). LP18, depending on ecological context, may influence butyrate-producing pathways either indirectly by modifying microbial composition or directly through metabolic interactions. Disrupted butyrate metabolism can significantly compromise intestinal function.

Butyrate is essential for the energy metabolism of colonocytes and supports epithelial integrity by enhancing the expression of tight junction proteins (Alberge et al., 2024). Its anti-inflammatory effects are achieved through the suppression of NF-κB signaling and the enhancement of regulatory immune responses and oxidative stress (Bakshi and Mishra, 2025). Therefore, decreased butyrate availability in SAP may generate a pathological environment marked by oxidative stress, epithelial apoptosis, and amplified inflammatory signaling. Although the significance of butyrate in maintaining intestinal homeostasis is well acknowledged, no research has examined whether LP18 influences butyrate metabolism during SAP or whether such regulation plays a role in intestinal injury.

Therefore, the current investigation seeks to clarify the function of *Lactobacillus paracasei* LP18 in SAP-related intestinal and inflammatory damage, with particular emphasis on butyrate metabolism mediated by the gut microbiota. Through the integration of 16S rRNA sequencing, targeted metabolomics, histological assessment, and molecular analyses, we investigated whether LP 18 could improve intestinal barrier dysfunction, reduce inflammatory responses, and decrease oxidative stress by increasing butyrate-producing bacteria and SCFA metabolic pathways. This study offers novel insights into the strain-specific effects of *Lactobacillus* in SAP and emphasizes butyrate metabolism as a key mechanistic pathway connecting microbial changes to intestinal injury. Our findings may inform more targeted probiotic interventions in clinical practice and facilitate the advancement of microbiota-focused strategies to alleviate gastrointestinal dysfunction associated with SAP.

## 2 Materials and methods

### 2.1 Preparation for LP18

LP18 was cultured in MRS (De Man, Rogosa, and Sharpe Broth) medium and incubated at 37 °C for 12 h. The LP18 strain was isolated via centrifugation at 4,000 rpm for 5 min at 4 °C, yielding a bacterial suspension of 1 × 10^8^ CFU/mL prepared with phosphate-buffered saline (PBS).

### 2.2 Animals and experimental design

Twenty-four male C57BL/6J rodents, 6 weeks old and weighing 16–18 g, were obtained from GemPharmatech Co., Ltd. The animal study was approved by the Animal Ethics Committee of The First Affiliated Hospital of Zhengzhou University (Approved No. ZZU-LAC2025032509). During the feeding phase, standard illumination conditions (12-h light-dark cycle), temperature (22 ± 2 °C), and sufficient food and water were provided daily. After a 7-day adaptation period, the rodents were arbitrarily allocated into three groups (*n* = 8): the control group (CON), the SAP group (SAP), and the LP18 group (LP18). The CON group and SAP group were administered 200 μL of PBS daily via gavage. The LP18 group was administered an intragastric dose of 200 μL of LP18 at a concentration of 1 × 10^8^ CFU/mL daily. Starting from day 28, mice in the SAP and LP18 groups were granted unrestricted access to injections of caerulein (Cae) and lipopolysaccharide (LPS). The SAP model was developed through intraperitoneal administration of Cae (300 μg/kg, NJPeptide, China, Cat. Pep03263) and LPS (10 μg/kg, MCE, USA, Cat. HY-D1056) in accordance with body weight. There were seven total administrations of Cae, with one injection given every hour. One hour after the final Cae injection, a single dose of LPS was administered. The control group was administered an identical volume of PBS injection. The initial administration of Cae was performed at 0 h. On the 29th day of the experiment, after a 10-h fast, the rodents were anesthetized with 3% isoflurane administered via inhalation for a duration of 2 min.

Pancreatic tissues were collected immediately after euthanasia. The abdominal cavity was rapidly opened under sterile conditions, and the pancreas was carefully dissected from surrounding tissues, including the stomach, spleen, duodenum, and mesentery, using fine forceps to avoid mechanical damage. The excised pancreas was gently rinsed in ice-cold phosphate-buffered saline (PBS) to remove blood and debris, blotted dry, and divided into portions for downstream analyses. For histological examination, pancreatic tissues were fixed in 4% paraformaldehyde at room temperature for 24 h, followed by paraffin embedding. For molecular analyses, tissues were snap-frozen in liquid nitrogen and stored at −80 °C until use. Blood was drawn from the cornea, centrifuged at 4 °C at 4,000 rpm for 15 min, and the serum was subsequently collected and stored at −80 °C until needed. The cecum contents and jejunum were collected in a sterile environment for subsequent microbiome sequencing. A section of the jejunum was immersed in 4% paraformaldehyde for subsequent histopathological examination, while the remaining specimens were cryopreserved in liquid nitrogen and stored at −80 °C.

### 2.3 Caco2 cell culture

Caco-2, a human colonic epithelial cell line, was cultured at 37 °C in a 5% (v/v) CO_2_ atmosphere using Dulbecco's Modified Eagle Medium (DMEM) supplemented with 10% (v/v) fetal bovine serum (FBS) and 1% (v/v) penicillin/streptomycin. A controlled environment with elevated humidity, maintained at 37 °C with a 5% carbon dioxide concentration, was employed for cell culture. Once the cells achieved 80% confluence, the culture medium was replaced every 2 days or the cells were harvested. The Caco2 cells were subsequently divided into three distinct groups: CON (treated solely with PBS), TNF-α (treated exclusively with TNF-α), and LP18 (treated with a combination of LP18 and TNF-α). The Caco2 cells in the control group were treated with phosphate-buffered saline (PBS), whereas the cells in the TNF-α and LP18 groups were exposed to 100 ng/mL TNF-α or 1 × 10^8^ CFU/mL LP18, respectively. In the experimental group designated as LP18, the Caco-2 cells were pretreated with a concentration of 1 × 10^8^ CFU/mL LP18 for a period of 24 h. Subsequently, the cells were subjected to a treatment with 100 ng/mL of TNF-α for a duration of 24 h. Prior to the administration of LP18 or TNF-α, each experimental group underwent two flushes with PBS.

### 2.4 Hematoxylin and eosin (H&E) staining

Intestinal morphological analysis entailed dehydrating jejunum tissue specimens through a sequential series of ethanol and xylene solutions, then fixing them in paraformaldehyde solution for 24 h at room temperature. The materials were subsequently processed into paraffin slabs. A 5 μm-thick cross-section was extracted from each specimen and stained with hematoxylin and eosin (Sigma-Aldrich, St. Louis, MO, USA). Following the administration of cedar oil, the tissues were examined and documented using a microscope (BX63, Olympus, Tokyo, Japan).

### 2.5 Biochemical assays

The concentrations ofIFN-γ, ALT, AST, D-LA, and DAO in the serum were quantified in accordance with the manufacturer's instructions (Nanjing Jiancheng Bioengineering Institute, Nanjing, China) using a spectrophotometer (SpectraMax M5, Molecular Devices, Sunnyvale, California, USA) in the laboratory.

### 2.6 Enzyme-linked immunosorbent assay (ELISA)

Mouse serum samples were collected, and concentrations of tumor necrosis factor-alpha (TNF-α), interleukin-1β (IL-1β), IL-2, IL-4, IL-6, and LPS were quantified using the same ELISA kits (Nanjing Jiancheng Bioengineering Institute, Nanjing, China). The absorbance was measured at 450 nm using a microplate reader.

### 2.7 Using chamber experiment

As previously reported, the Ussing chamber experiment was conducted to assess jejunum permeability (Hu et al., 2013). The proximal segment of the jejunum was excised, treated with Ringer's solution, and secured using a Ussing chamber system (VCC MC6, San Diego, CA). Following equilibration, the transendothelial electrical resistance (TER, Ω·cm^2^) was assessed at 15-min intervals over a duration of 2 h. The TER value of a caco2 cells was determined by calculating the mean of the data. On the mucosal surface, FITC-labeled dextran (FD4, Sigma-Aldrich, Saint Louis, MO, USA) was administered. FD4 flux from the mucosal to the serosal side was assessed at 30-min intervals over a 90-min period utilizing a multifunction microplate reader (Bio-Tek, Flx800, USA).

### 2.8 RNA extraction and RTqPCR

Total RNA was isolated from jejunum and Caco2 tissues employing TRIZOL reagent (TaKaRa, Japan). RNA purity and integrity were evaluated using spectrophotometry (SpectraMax M5, Molecular Devices, USA), with A260/A280 ratios maintained within the range of 1.8–2.0. cDNA synthesis was performed using the PrimeScript II First Strand cDNA Synthesis Kit (Vazyme, Nanjing, Jiangsu, China). For RT-qPCR analysis, reactions were conducted in triplicate on 96-well plates utilizing the StepOne Plus Real-Time PCR System (Applied Biosystems, Carlsbad, CA, USA) and the HiScript II One Step RT-qPCR SYBR Green Kit (Vazyme, Nanjing, Jiangsu, China). Primers designed using NCBI Primer-Blast are provided in Table S3. Gene expression levels were normalized to β-actin and quantified utilizing the 2–ΔΔCt method.

### 2.9 Western blotting

Jejunum tissue lysates prepared with RIPA buffer were subjected to BCA protein quantitation (Sigma, Saint Louis, MO, USA). The protease inhibitor PMSF was freshly added to the RIPA buffer immediately before use according to the manufacturer's instructions. Samples were incubated on ice for 30 min with intermittent vortexing and subsequently centrifuged at 12,000 × *g* for 15 min at 4 °C. The supernatants were collected, and protein concentrations were determined using a BCA assay prior to downstream analyses. Equal protein concentrations were subjected to 12% SDS-PAGE and subsequently deposited onto PVDF membranes (Millipore, MA, USA) through electroblotting. Membranes were blocked with 5% skim milk for 2 h at 26 °C and subsequently incubated overnight at 4 °C with primary antibodies targeting claudin-1, Occludin, p-NF-κB p65, NF-κB p65 and TRAF6 and β-actin (Cell Signaling Technology, MA, USA). Following five TBST washes, HRP-conjugated secondary antibodies were applied for 2 h at 26 °C. Protein bands were detected using a Millipore chemiluminescent HRP substrate kit (MA, USA) and imaged with a Tanon system (China). Band intensities quantified using ImageJ software, with protein levels normalized to β-actin.

### 2.10 Intestinal microbial DNA extraction and high-throughput sequencing

Genomic DNA from microbes was isolated from rodent cecum contents under sterile conditions employing the TIANamp Stool DNA Kit (Tiangen, Beijing, China). DNA integrity was confirmed through spectrophotometric analysis and agarose gel electrophoresis. Amplification of the 16S rRNA V3–V4 region was performed using primers 338F (5′-ACTCCTACGGGAGGCAGCAG-3′) and 806R (5′-GGACTACHVGGGTWTCTAAT-3′), with nuclease-free water serving as a negative control. PCR products were subjected to electrophoresis in a 2% agarose gel. DADA2 (version 1.8) and QIIME2 (https://qiime2.org/) were utilized to denoise the data, followed by the excision of chimeric sequences using VSEARCH (version 2.15.0). Operational taxonomic unit (OTU) tables were generated from quality-filtered readings, employing SILVA-based taxonomy assignment via the RDP classifier (version 2.2). Alpha and beta diversity assessments employed unweighted UniFrac distance metrics. Differential abundance analysis at the phylum/genus level and functional predictions based on PICRUSt2 were conducted.

### 2.11 Untargeted metabolomic analysis

Serum samples (100 μL) were processed according to established protocols (Gao et al., 2016): combined with 400 μL of 80% methanol in EP tubes, vortexed, and centrifuged at 4 °C at 15,000 g for 20 min. Supernatants were diluted with mass spectrometry-grade water to achieve a methanol concentration of 53%. Pooled quality control samples derived from equal aliquots ensured analytical consistency. Chromatographic separation was conducted using a Vanquish UHPLC system (Thermo Fisher, MA, USA) equipped with a Hypesil Gold column (100 × 2.1 mm, 1.9 μm) in both positive and negative ion modes. Positive mode conditions: mobile phase A = 0.1% formic acid, B = methanol; negative mode conditions: both phases = methanol (5 mM ammonium acetate in A, pH 9.0). Column parameters: discharge rate of 0.2 mL/min, temperature set to 40 °C. Mass spectrometry was conducted using a Q Exactive™ HF instrument (Thermo Fisher, MA, USA) with a scan range of 100–1,500 m/z. ESI settings: spray voltage 3.5 kV, sheath gas at 35 kPa, auxiliary gas at 10 L/min, capillary temperature 320 °C, S-lens RF 60, auxiliary heater at 350 °C.

### 2.12 Detection of SCFA levels

Previous reports (Zhang et al., 2024) were used to prepare the sample. The following parameters were employed to quantify the levels of SCFA: a flow rate of 2 mL/min, an initial column temperature of 90 °C, a duration of 6 min, a temperature ramp of 10 °C/min to 200 °C, followed by an additional 6-min hold. The gas chromatograph employed was an Agilent 7890, located in Palo Alto, California, USA. The carrier gas has a flow rate of 2 mL/min. With a hydrogen flow rate of 30 mL/min and an air flow rate of 300 mL/min, the FID detector is maintained at a temperature of 250 °C.

### 2.13 Statistical analysis

Statistical analyses were conducted using SPSS 20.0 (IBM, USA) for one-way ANOVA with Tukey's *post hoc* test to perform multiple comparisons. Data presented as mean ± SEM; *P* < 0.05 denotes statistical significance. Graphs produced using GraphPad Prism 8.4.2.

## 3 Result

### 3.1 LP18 alleviated the pancreatic injury in the SAP mice

**Figure 1:**
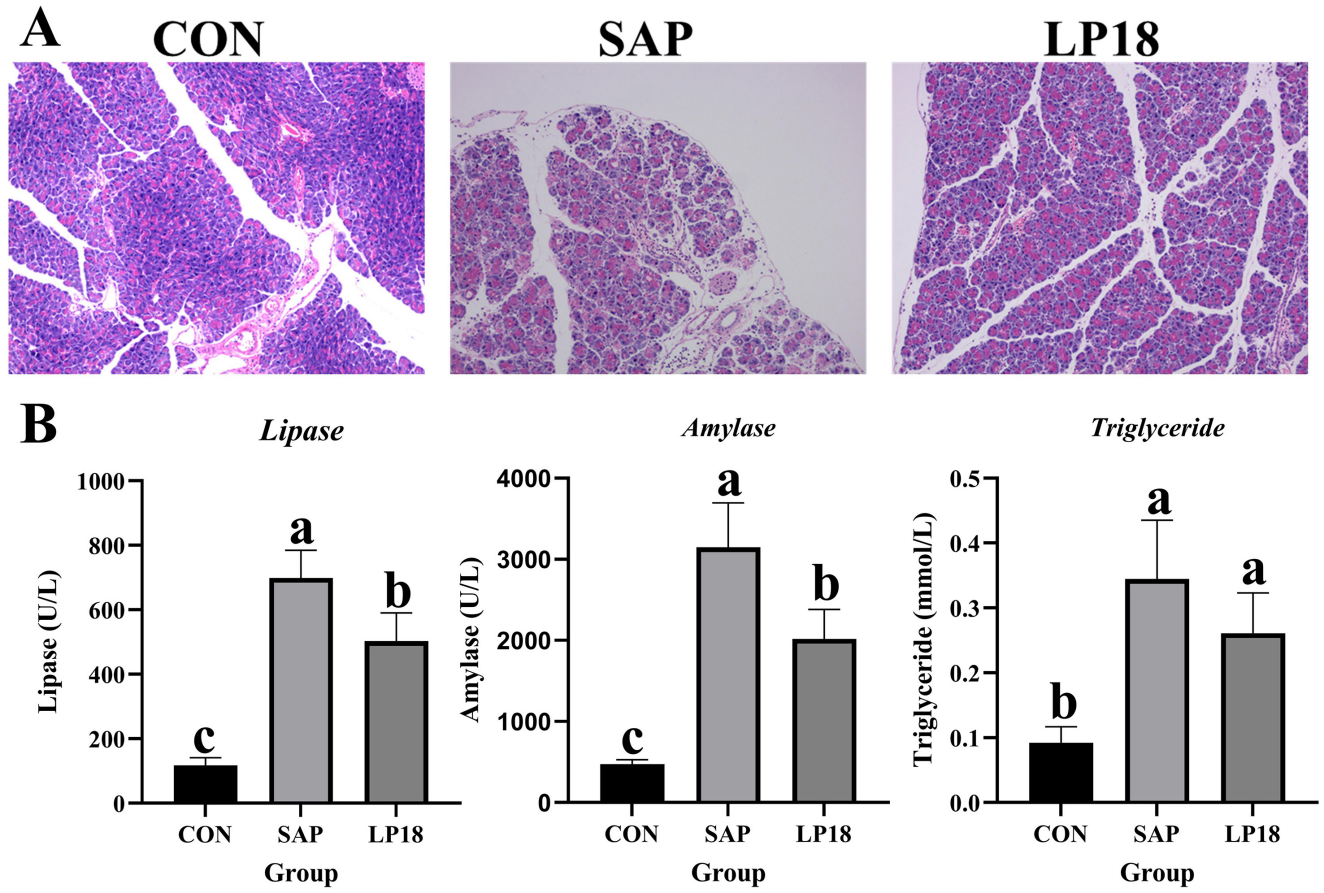
Histological analysis and serum biochemical parameters in CON, SAP, and LP18 groups. **(A)** Representative H&E-stained pancreatic sections from the CON, SAP, and LP18 groups. **(B)** Serum levels of lipase, amylase, and triglycerides in the three groups. Data represent mean ± SD (*n* = 4). Distinct superscript letters ^(a,b,c)^ denote statistically significant intergroup differences (*P* < 0.05).

Comparing with the normal pancreatic morphology in the control group, the pancreas tissues showed typical damage in the SAP group, including extensive edema, inflammatory infiltration and vacuolation of acinar cells. In contrast to the morphological disruption in the SAP group, LP18 administration markedly alleviated the histological injury in the pancreas, as reflected by reduced stromal edema and preservation of acinar integrity (Figure 1A). In addition, the LP18 supplementation also significantly (*P* < 0.05) restored the pancreatic exocrine function, with a notable (*P* < 0.05) decrease in the levels of enzyme, lipase and triglyceride comparing with the SAP group (Figure 1B).

### 3.2 LP18 preserved intestinal morphology and improved barrier integrity in SAP mice

**Figure 2:**
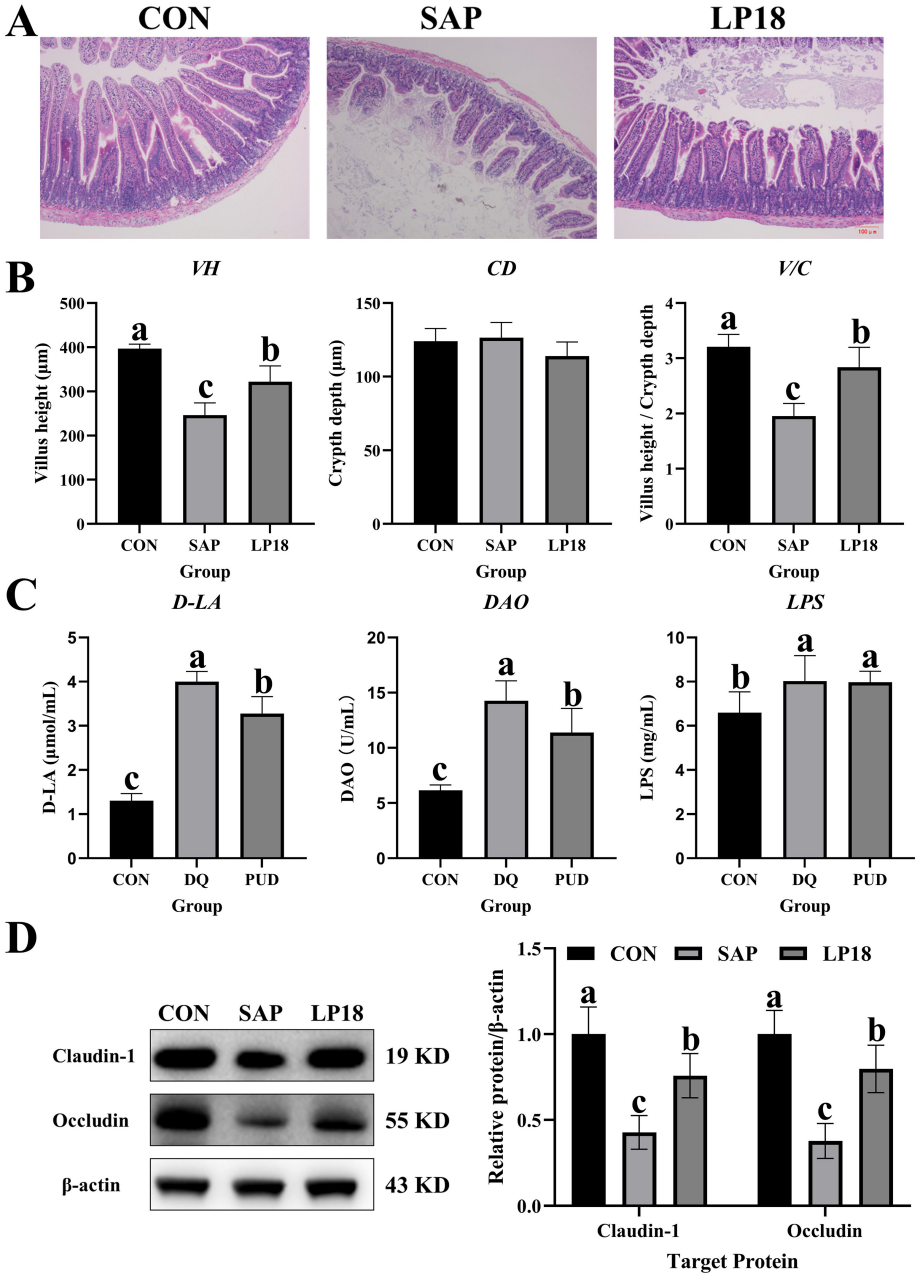
Intestinal morphology, permeability indicators, and tight junction protein expression in CON, SAP, and LP18 groups. **(A)** Representative H&E-stained small-intestinal sections. **(B)** Villus height, crypt depth, and villus height/crypt depth ratio. **(C)** Serum levels of D-lactic acid (D-LA), diamine oxidase (DAO), and lipopolysaccharide (LPS). **(D)** Protein expression of claudin-1 and occludin and their quantitative analysis. Data represent mean ± SD (*n* = 4). Distinct superscript letters ^(a,b,c)^ denote statistically significant intergroup differences (*P* < 0.05).

Histological analysis of the small intestine revealed marked villus atrophy and mucosal injury in the SAP group comparing with the control group (Figure 2A). However, the LP18 supplementation ameliorated the villus structure and improved overall mucosal morphology. Quantitative measurements demonstrated a substantial reduction in villus height and villus height-to-crypt depth ratio in SAP mice (Figure 2B), whereas LP18 treatment significantly (*P* < 0.05) increased both parameters relative to the SAP group, indicating improved epithelial architecture. To further evaluate intestinal barrier permeability, the circulating levels of D-lactic acid (D-LA), diamine oxidase (DAO), and lipopolysaccharide (LPS) were measured (Figure 2C). Consistent with impaired barrier function, these molecules were significantly increased in the SAP group, while LP18 treatment effectively mitigated their elevation (*P* < 0.05), suggesting partial restoration of barrier integrity. Protein expression of the tight junction markers claudin-1 and occludin was examined by Western blotting (Figure 2D). SAP mice exhibited notable (*P* < 0.05) reductions in both proteins, whereas LP18 treatment significantly (*P* < 0.05) increased their expression relative to SAP, indicating improved tight junction maintenance.

### 3.3 LP18 attenuated SAP-induced hepatic injury and modulates systemic inflammatory cytokine profiles

**Figure 3:**
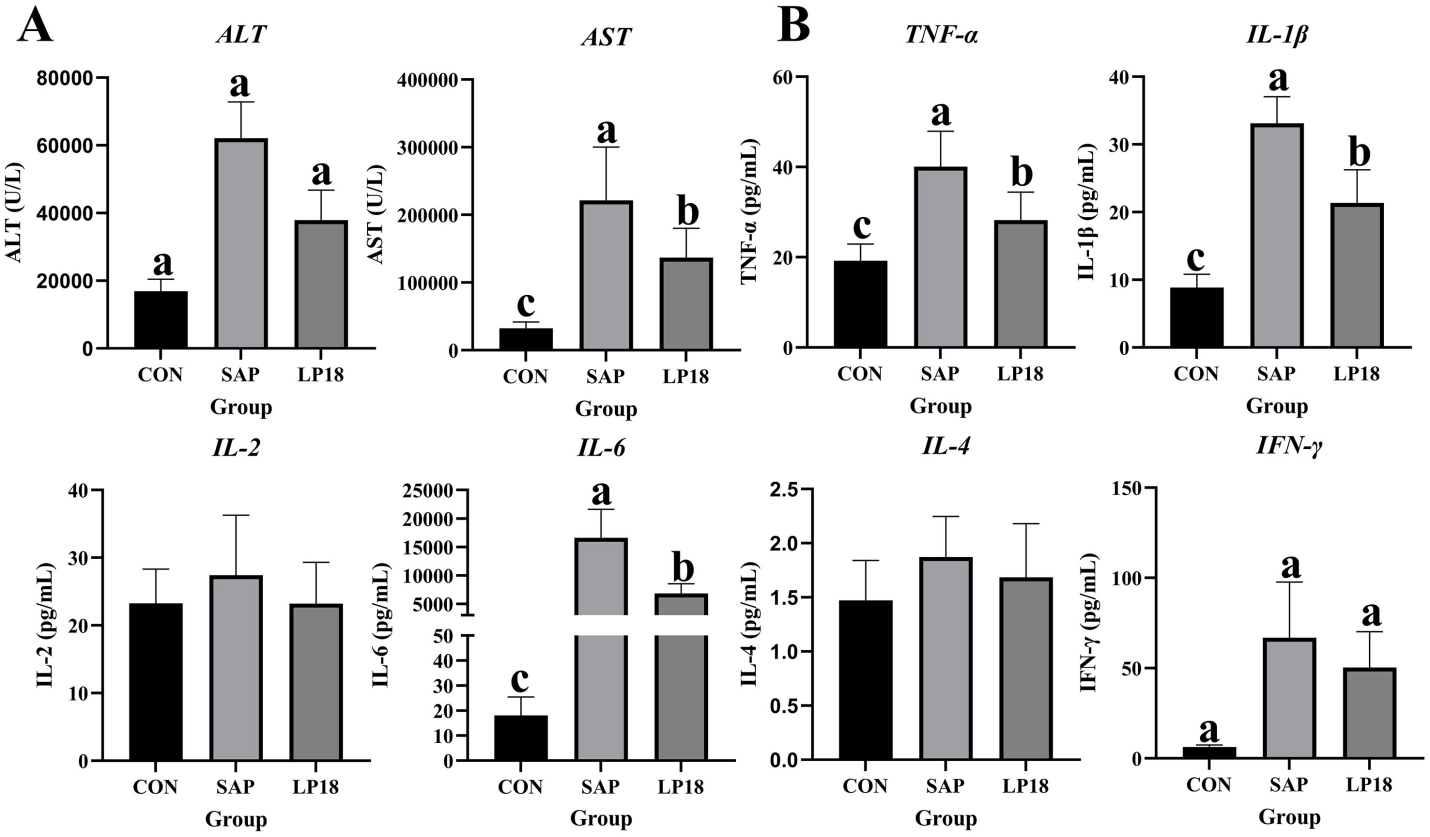
The levels of key inflammatory cytokine and liver enzymes in serum of mice in CON, SAP, and LP18 groups. **(A)** Serum level of ALT and AST. **(B)** Serum levels of TNF-α, IL-1β, IL-2, IL-6, IL-4, and IFN-γ. Data represent mean ± SD (*n* = 4). Distinct superscript letters ^(a,b,c)^ denote statistically significant intergroup differences (*P* < 0.05). ALT: alanine transaminase; AST: aspartate aminotransferase.

To evaluate the effect of LP18 on the systemic inflammation, the levels of inflammatory factors in serum were assessed (Figures 3A, B). Compared with the SAP group, the levels of liver enzymes were significantly decreased in the LP18 group, indicating that LP18 exerts a protective effect on the liver (Figure 3A). The concentrations of key proinflammatory cytokines, including tumor necrosis factor-alpha (TNF-α), interleukin-1 beta (IL-1β) and interleukin-6 (IL-6), were significantly (*P* < 0.05) increased in the SAP group. In contrast, LP18 treatment significantly (*P* < 0.05) reduced the upregulated levels of these cytokines compared with the SAP group (Figure 3B).

### 3.4 LP18 modulated gut microbial diversity and community structure in SAP mice

**Figure 4:**
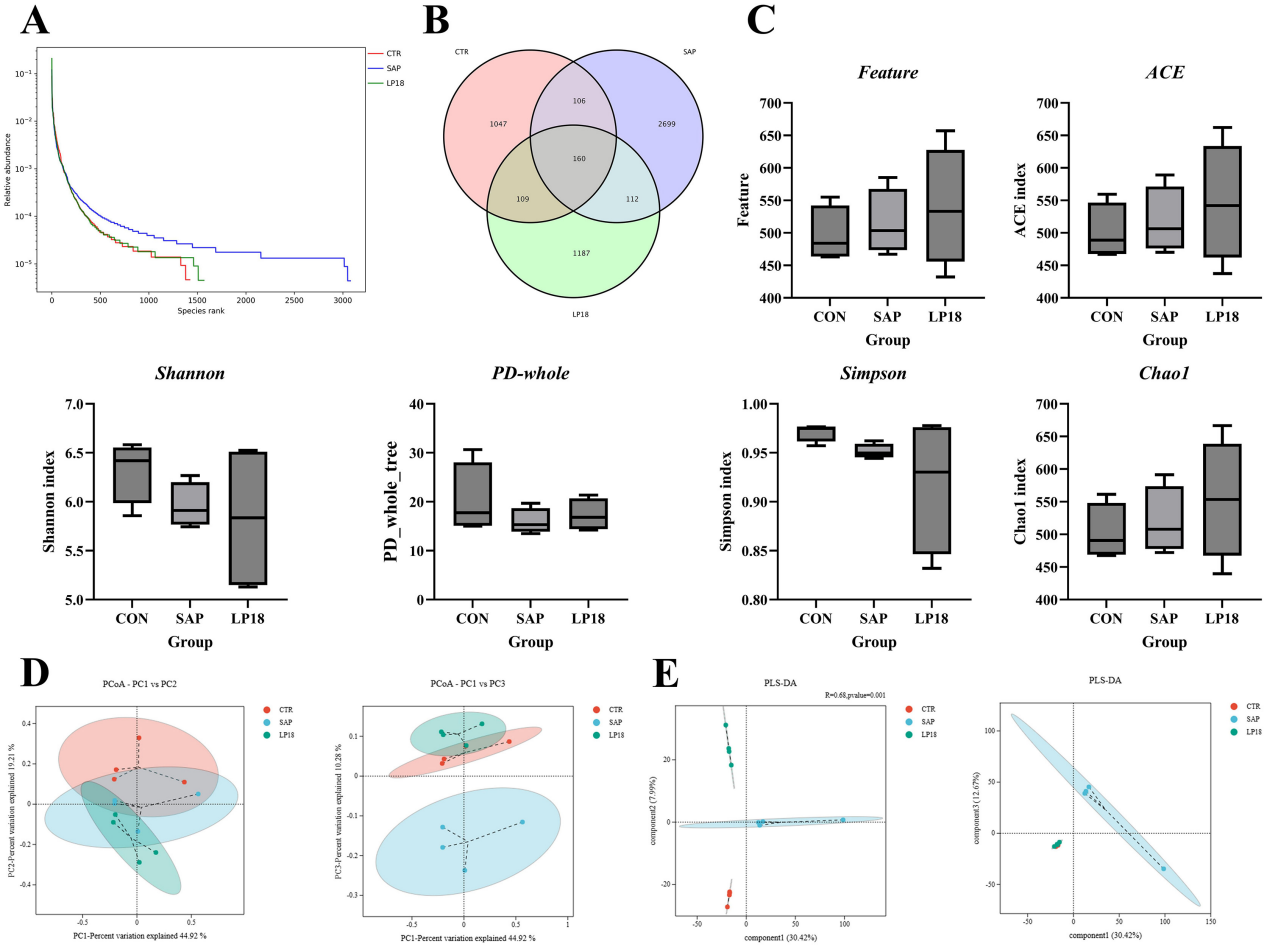
Gut microbiota diversity and community structure in CON, SAP, and LP18 groups. **(A)** Rank–abundance curves. **(B)** Venn diagram of shared and unique OTUs. **(C)** α-diversity indices (Shannon, PD-whole tree, Simpson, ACE, Chao1, and Feature count). **(D)** Principal coordinate analysis (PCoA) plots. **(E)** Partial least squares discriminant analysis (PLS-DA) plots. Data represent mean ± SD (*n* = 4). Distinct superscript letters ^(a,b,c)^ denote statistically significant intergroup differences (*P* < 0.05).

The overall distribution of microbial abundance across ranked species is shown in Figure 4A. The relative abundance curve was altered in the SAP group compared with the control group, whereas the LP18 supplementation led to a significant change in the distribution pattern. Venn analysis demonstrated both shared and unique operational taxonomic units (OTUs) among the three groups (Figure 4B). α-diversity indices, including Feature count, ACE, Simpson, and Chao1, were subsequently assessed (Figure 4C). SAP mice displayed reduced Shannon and PD-whole tree indices relative to the control group, indicating decreased microbial richness and phylogenetic diversity. LP18 supplementation increased Shannon and PD-whole tree values compared with the SAP group. Similarly, Feature count, ACE, and Chao1 indices showed higher values in LP18-treated mice relative to SAP, whereas Simpson index remained relatively stable across groups. β-diversity analyses illustrated clear separation of microbial community structures among groups. Principal coordinate analysis (PCoA) plots showed distinct clustering of CON, SAP, and LP18 groups based on PC1, PC2, and PC3 (Figure 4D). Partial least squares discriminant analysis (PLS-DA) further emphasized these separations, demonstrating distinct microbial community profiles in each group (Figure 4E).

### 3.5 LP18 altered key differential gut taxa identified by LEfSe analysis in SAP mice

**Figure 5:**
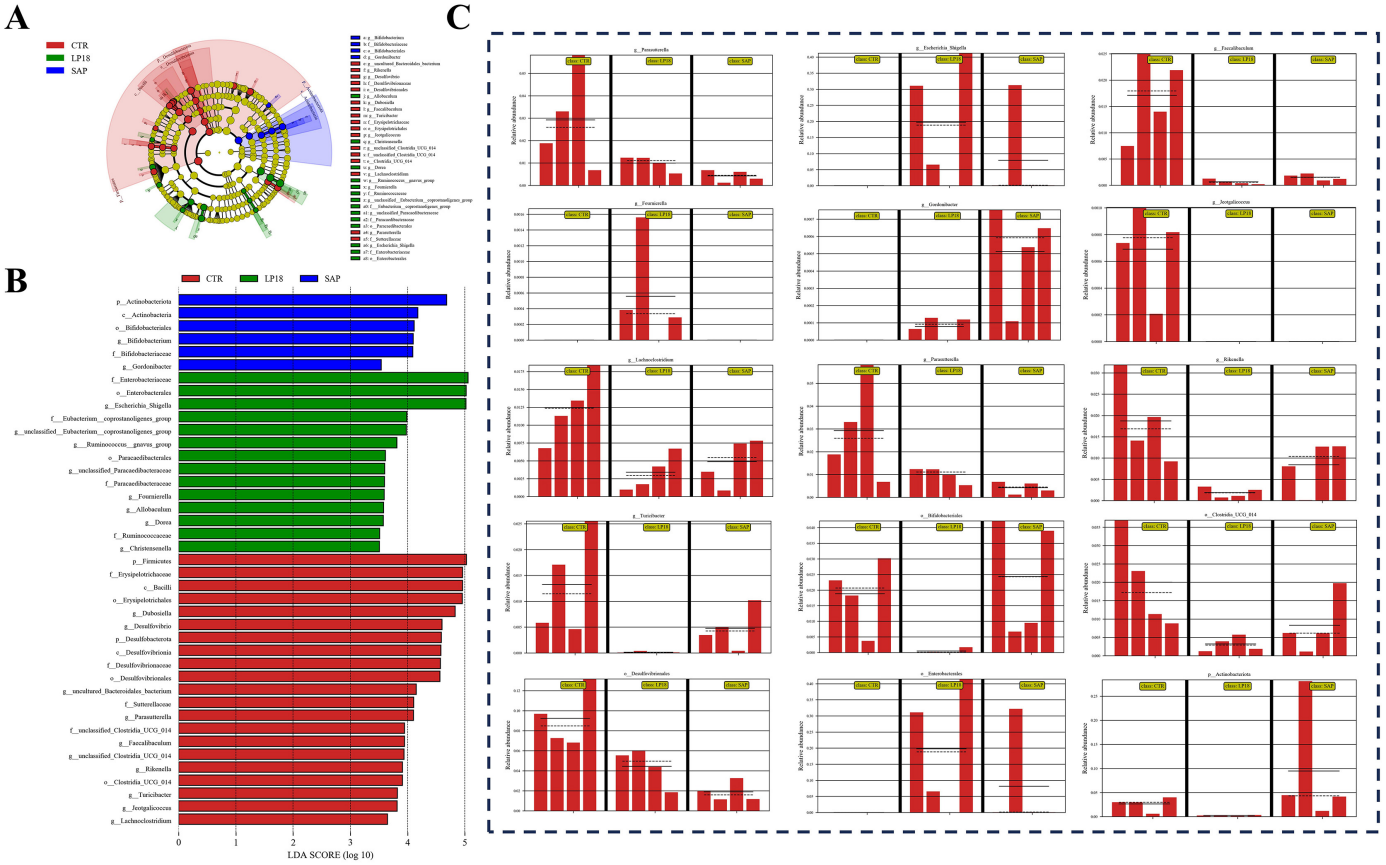
LEfSe-based identification of discriminatory taxa among CON, SAP, and LP18 groups. **(A)** LEfSe cladogram illustrating differentially enriched taxa across groups. **(B)** LDA score plots showing taxa with significant discriminative contributions. **(C)** Relative abundance of representative differential genera identified by LEfSe.

To further identify specific taxa contributing to microbial differences among groups, LEfSe analysis was performed. The cladogram (Figure 5A) illustrated distinct bacterial enrichments across the CON, SAP, and LP18 groups. SAP mice exhibited strong enrichment of taxa belonging to *Actinobacteriota*, including the class *Actinobacteria*, order *Bifidobacteriales*, family *Bifidobacteriaceae*, and genus *Bifidobacterium*. LP18 treatment was associated with increased abundance of several taxa primarily within *Firmicutes*, including *Allobaculum, Dubosiella, Fournierella, Christensenella*, and members of *Ruminococcaceae*. In contrast, the control group displayed enrichment across a broader set of bacterial lineages, such as *Turicibacter, Lachnoclostridium, Parasutterella, Desulfovibrio*, and taxa associated with *Clostridia*- and *Desulfobacterota*-related families.

Consistent with the cladogram, LDA score analysis revealed clear group-specific discriminatory taxa (Figure 5B). SAP mice showed high LDA scores for *Actinobacteriota*-associated taxa, whereas the LP18 group was characterized by differential enrichment of multiple *Firmicutes*-derived genera. Control mice demonstrated enrichment of several genera from *Firmicutes, Proteobacteria*, and *Desulfobacterota*.

Relative abundance plots of representative differential genera are presented in Figure 5C. These taxa exhibited distinct distribution patterns among the three groups, reflecting the group-specific enrichment identified by LEfSe analysis. Genera enriched in SAP mice, such as *Bifidobacterium, Gordonibacter*, and members of *Bifidobacteriales*, were lower or absent in LP18-treated mice. Conversely, LP18 supplementation increased the abundance of several *Firmicutes*-associated genera that were less represented in SAP mice.

### 3.6 LP18 remodeled metabolic profiles in the SAP mice as revealed by multivariate and differential metabolite analyses

**Figure 6:**
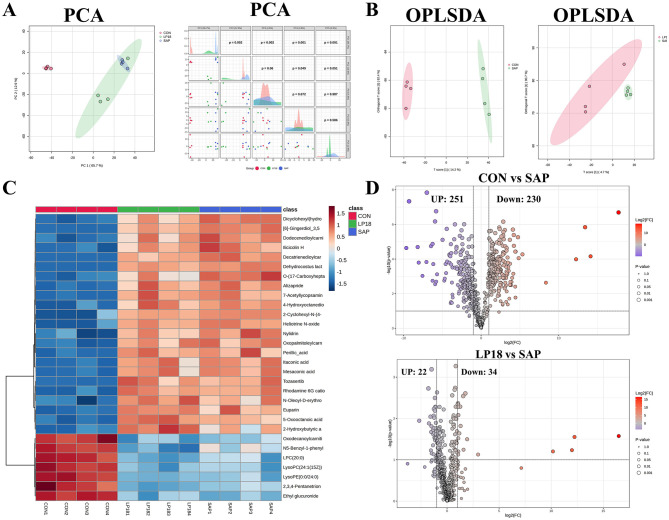
Multivariate and differential metabolite analyses in CON, SAP, and LP18 groups. **(A)** PCA score plots and pairwise PCA component distributions. **(B)** OPLS-DA plots distinguishing group-specific metabolic profiles. **(C)** Heatmap of significantly altered metabolites across samples. **(D)** Volcano plots showing differential metabolites in CON vs SAP and LP18 vs SAP comparisons.

Principal component analysis (PCA) showed clear separation between CON and SAP groups, with LP18 displaying a distinct clustering pattern (Figure 6A). The PCA scatter plots and pairwise component distributions demonstrated that there were marked alterations in global metabolic profiles between the SAP and CON groups. LP18-treated mice exhibited a shift away from the SAP cluster, indicating a partial restoration of metabolic patterns. Orthogonal partial least squares discriminant analysis (OPLS-DA) further revealed distinct metabolic signatures among groups (Figure 6B). Clear group separation was observed between CON and SAP, as well as between LP18 and SAP groups, confirming systematic metabolic differences occurred in the SAP mice but modified by LP18.

A heatmap of significantly altered metabolites across samples (Figure 6C) demonstrated marked clustering of metabolic features by group. CON and SAP groups exhibited divergent profiles, while LP18 samples showed an intermediate pattern, characterized by distinct changes in multiple metabolite classes, including lipids, organic acids, and bioactive small molecules. Volcano plot analysis identified numerous differentially expressed metabolites (Figure 6D). A total of 251 metabolites were upregulated and 230 were downregulated in SAP compared with CON. In contrast, comparison between LP18 and SAP identified 22 upregulated and 34 downregulated metabolites, indicating a relatively moderate but targeted metabolic shift following LP18 treatment.

### 3.7 LP18 modulated key metabolic pathways by KEGG enrichment analysis in SAP mice

**Figure 7:**
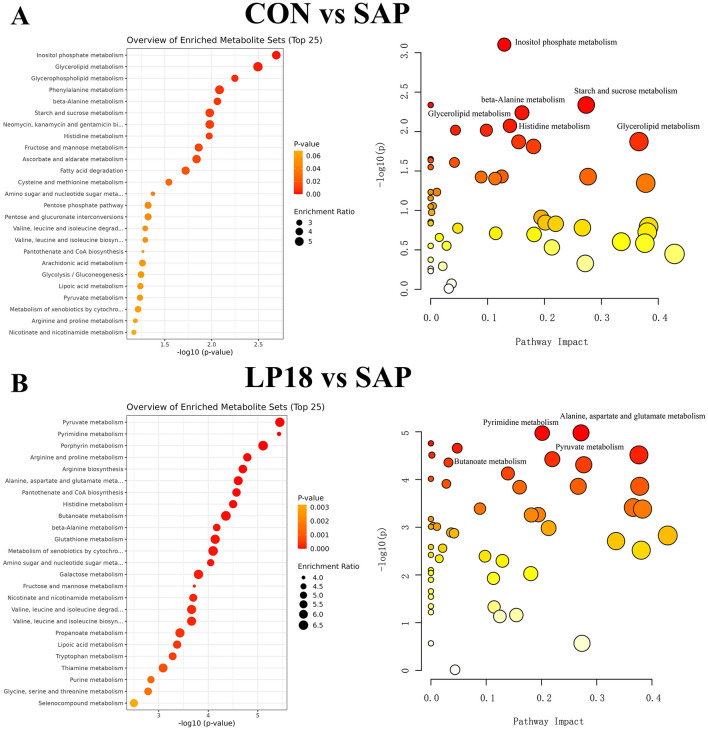
KEGG pathway enrichment analysis based on differential metabolites. **(A)** Top 25 enriched metabolic pathways and corresponding bubble plot for CON vs. SAP. **(B)** Top 25 enriched metabolic pathways and corresponding bubble plot for LP18 vs. SAP.

KEGG pathway enrichment analysis was performed to identify metabolic pathways associated with differential metabolites. As shown in Figure 7A, comparison between CON and SAP groups revealed significant enrichment of multiple pathways, including inositol phosphate metabolism, glycerolipid metabolism, glycerophospholipid metabolism, phenylalanine metabolism, and β-alanine metabolism. Bubble plots further indicated that several of these pathways exhibited high pathway impact values, reflecting substantial metabolic perturbation induced by SAP.

Comparison of LP18 and SAP groups identified a distinct set of enriched pathways (Figure 7B). Metabolic pathways such as pyruvate metabolism, pyrimidine metabolism, porphyrin metabolism, arginine and proline metabolism, and alanine, aspartate, and glutamate metabolism were significantly enriched. Pathways with high impact scores included pyruvate metabolism, arginine biosynthesis, and amino acid-related metabolic modules.

### 3.8 LP18 modulated SCFA profiles altered in the SAP mice

**Figure 8:**
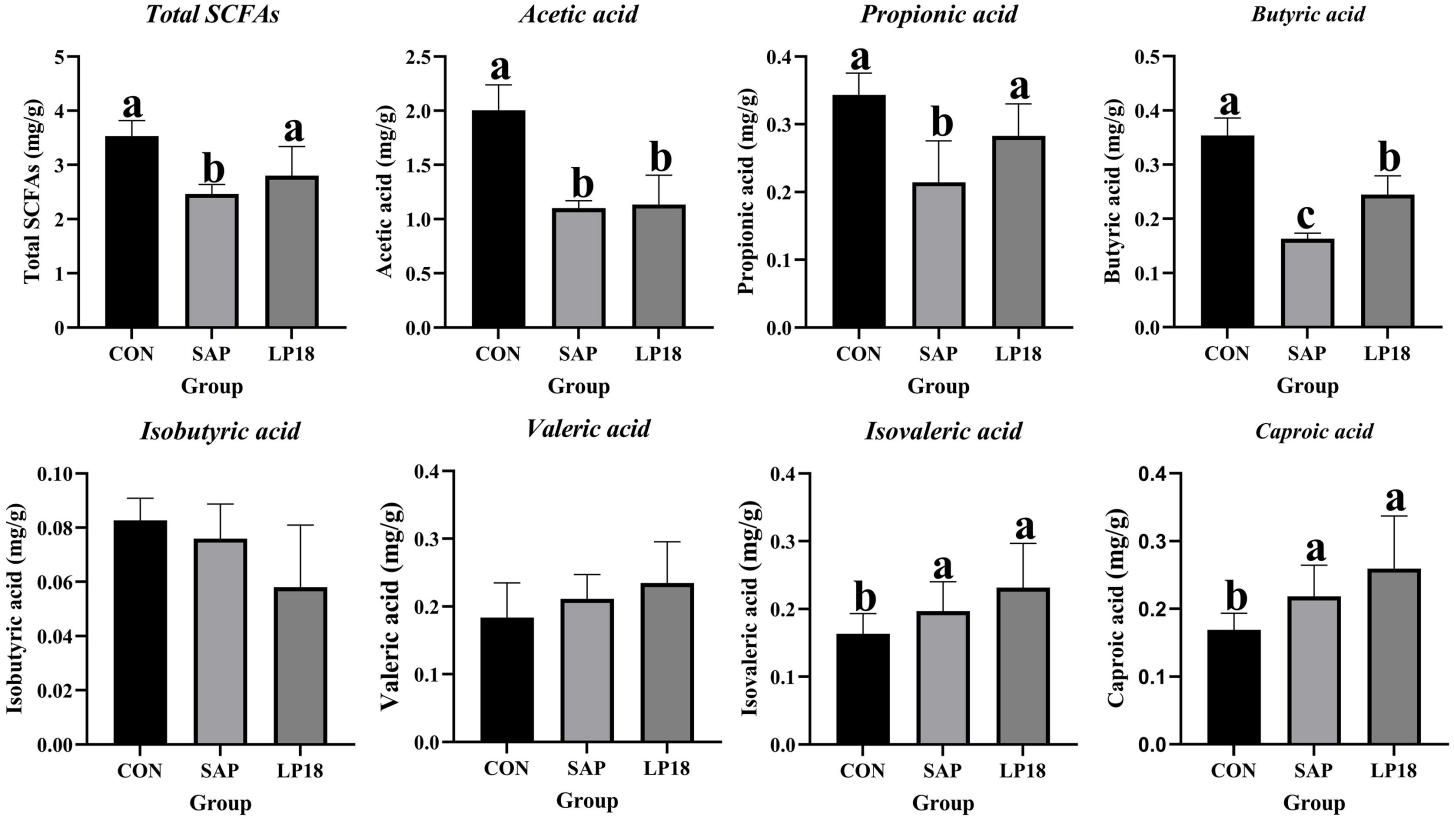
Short-chain fatty acid (SCFA) concentrations in CON, SAP, and LP18 groups. Total SCFAs, acetic acid, propionic acid, butyric acid, isobutyric acid, valeric acid, isovaleric acid, and caproic acid. Data represent mean ± SD (*n* = 4). Distinct superscript letters ^(a,b,c)^ denote statistically significant intergroup differences (*P* < 0.05).

Short-chain fatty acids (SCFAs) were measured to evaluate microbial metabolic output among groups (Figure 8). SAP mice exhibited a reduction in total SCFA levels compared with the control group. LP18 supplementation increased total SCFAs relative to SAP group, resulting in levels comparable to controls. Among individual SCFAs, acetic acid and propionic acid were both decreased (*P*<*0.05*) in the SAP mice relative to controls, while LP18 treatment increased their concentrations compared with the SAP group. Butyric acid was markedly (*P*<*0.05*) reduced in the SAP mice but partially restored in the LP18 group. Isobutyric acid and valeric acid showed no significant differences among groups. Levels of isovaleric acid were higher in SAP and LP18 groups than in controls. Caproic acid increased in both SAP and LP18 groups relative to controls.

### 3.9 LP18 modulated SCFA receptor expression and downstream NF-κB Signaling in SAP mice

**Figure 9:**
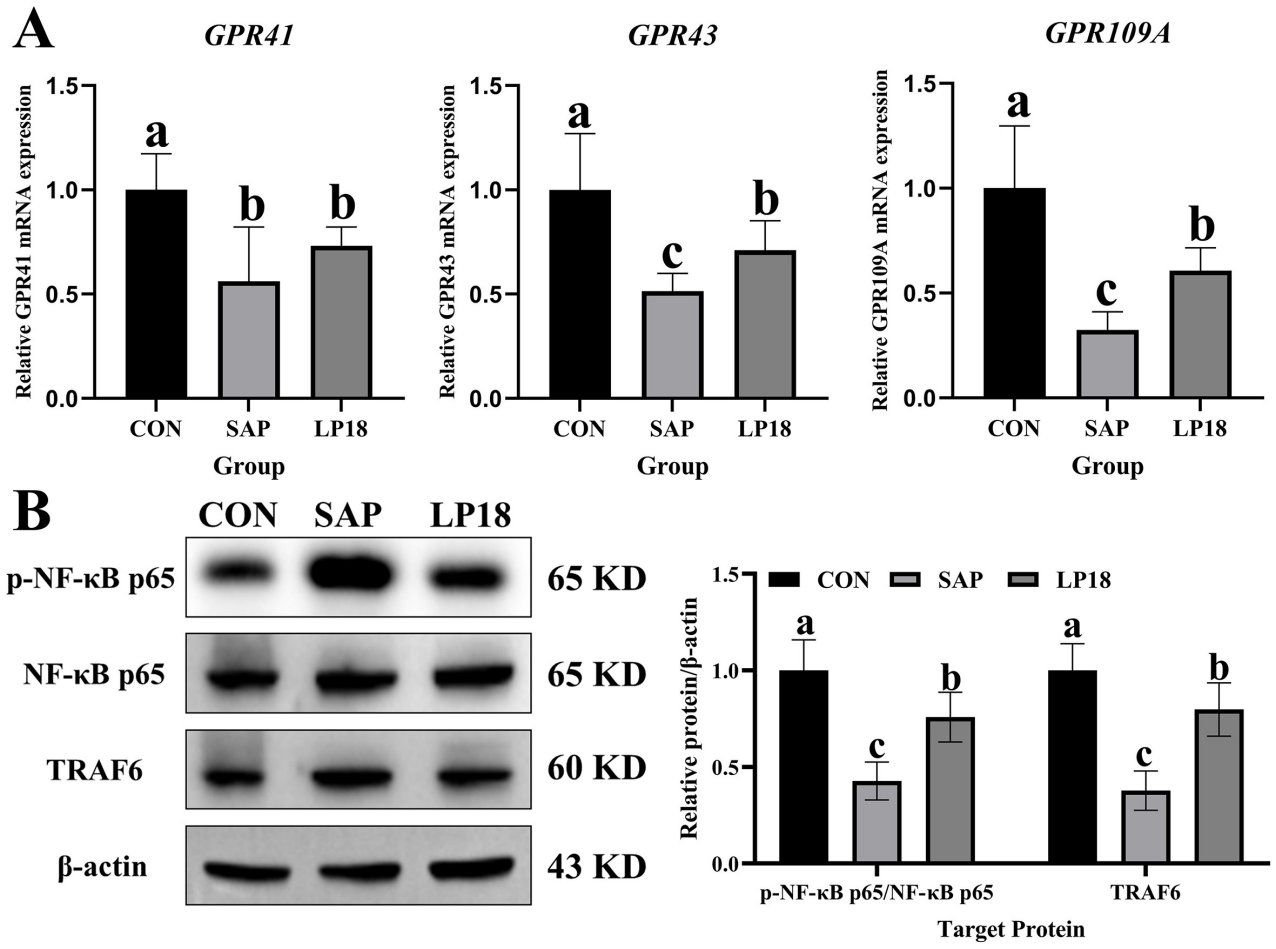
Expression of SCFA receptors and NF-κB signaling proteins in CON, SAP, and LP18 groups. **(A)** mRNA expression levels of *GRP41, GRP43*, and *GRP109A*. **(B)** Western blot analysis of p-NF-κB p65, NF-κB p65, and TRAF6, with corresponding quantitative analysis. Data represent mean ± SD (*n* = 4). Distinct superscript letters ^(a,b,c)^ denote statistically significant intergroup differences (*P* < 0.05).

Expression levels of SCFA receptors GRP41, GRP43, and GRP109A were further evaluated among these three groups (Figure 9A). The relative mRNA expression levels of *GRP41, GRP43*, and *GRP109A* were significantly reduced in the SAP group compared with the control group, whereas levels of *GRP43* and *GRP109A* were markedly restored after the LP18 treatment. However, the expression level of GRP41 still remained lower in both SAP and LP18 groups than in controls. To further examine the downstream inflammatory signaling, protein levels of phosphorylated NF-κB p65 (p-NF-κB p65), total NF-κB p65 (t-NF-κB p65), and TRAF6 were analyzed (Figure 9B). Compared with the control group, the ratio of p-NF-κB p65 to t-NF-κB p65 and the relative expression level of TRAF6 protein were increased in the SAP mice. However, both these two levels were significantly reduced after LP18 treatment, indicating partial attenuation of the activation of NF-κB pathway induced by SAP.

### 3.10 LP18 attenuated TNF-α induced injury in intestinal epithelial cells *in vitro*

**Figure 10:**
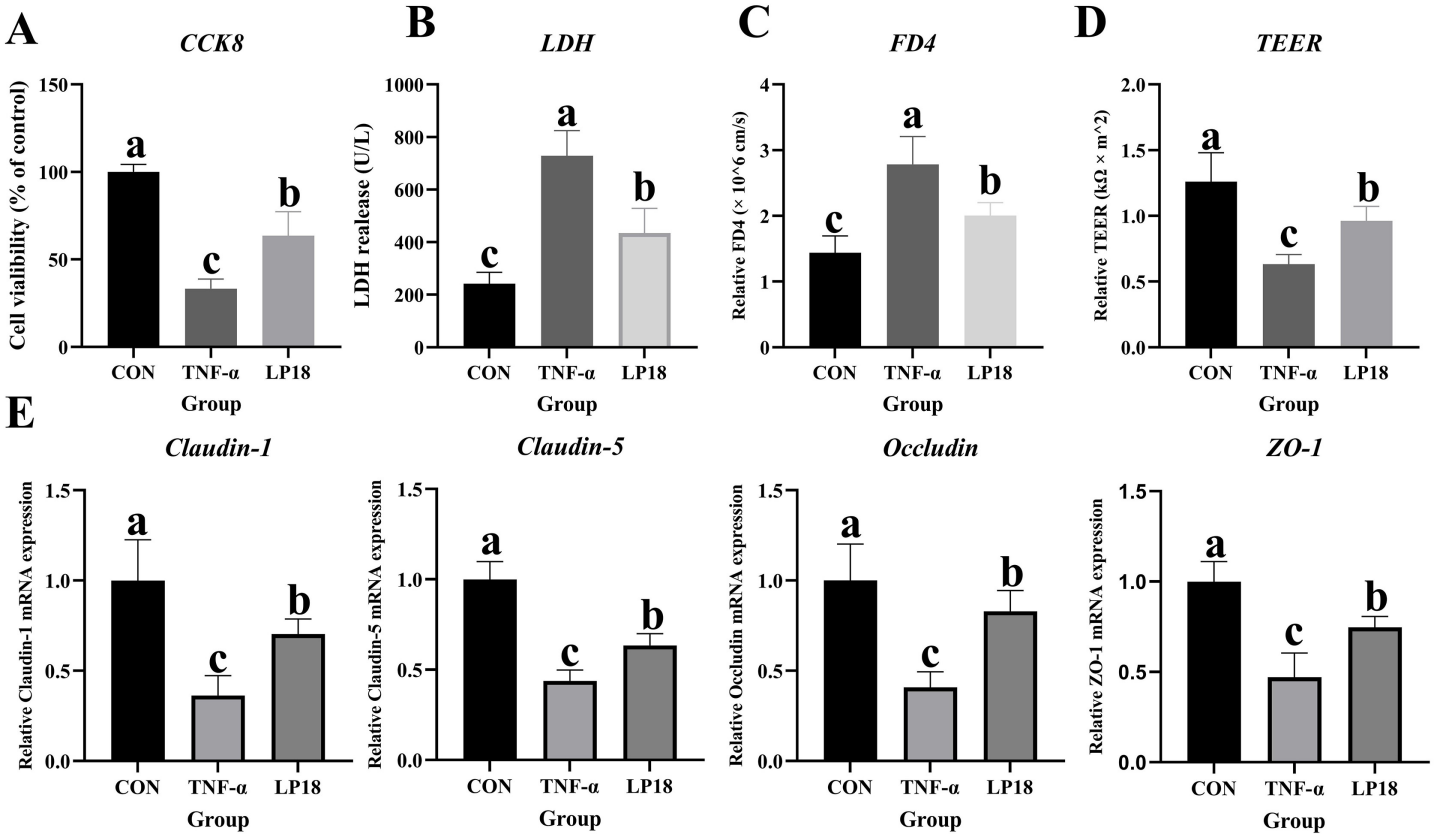
Effects of LP18 on TNF-α-induced epithelial injury *in vitro*. **(A)** Cell viability. **(B)** LDH release. **(C)** FD4 permeability. **(D)** TEER. **(E)** mRNA expression of Claudin-1, Claudin-5, Occludin, and ZO-1. Data represent mean ± SD (*n* = 4). Distinct superscript letters ^(a,b,c)^ denote statistically significant intergroup differences (*P* < 0.05).

Cell viability, membrane damage, epithelial permeability, and tight junction-related gene expression were evaluated in TNF-αstimulated epithelial cells (Figure 10). TNF-α markedly reduced cell viability compared with the control group, whereas LP18 treatment partially restored viability (Figure 10A). LDH release was significantly increased in TNF-α-treated cells, indicating enhanced cellular damage; LP18 reduced LDH release relative to TNF-α (Figure 10B). Permeability measurements showed that TNF-α increased FD4 flux while decreasing TEER values, demonstrating impaired barrier integrity (Figures 10C, D). LP18 treatment decreased FD4 flux and partially restored TEER compared with TNF-α, indicating improved epithelial barrier function. Expression of tight junction-related genes, including *Claudin-1, Claudin-5, Occludin*, and *ZO-1*, was significantly downregulated in TNF-α-treated cells (Figure 10E). LP18 supplementation upregulated all four genes relative to TNF-α, although levels remained lower than those in untreated control cells.

### 3.11 LP18 modulated inflammatory cytokine expression and MyD88-NF-κB pathway activation in TNF-α stimulated cells

**Figure 11:**
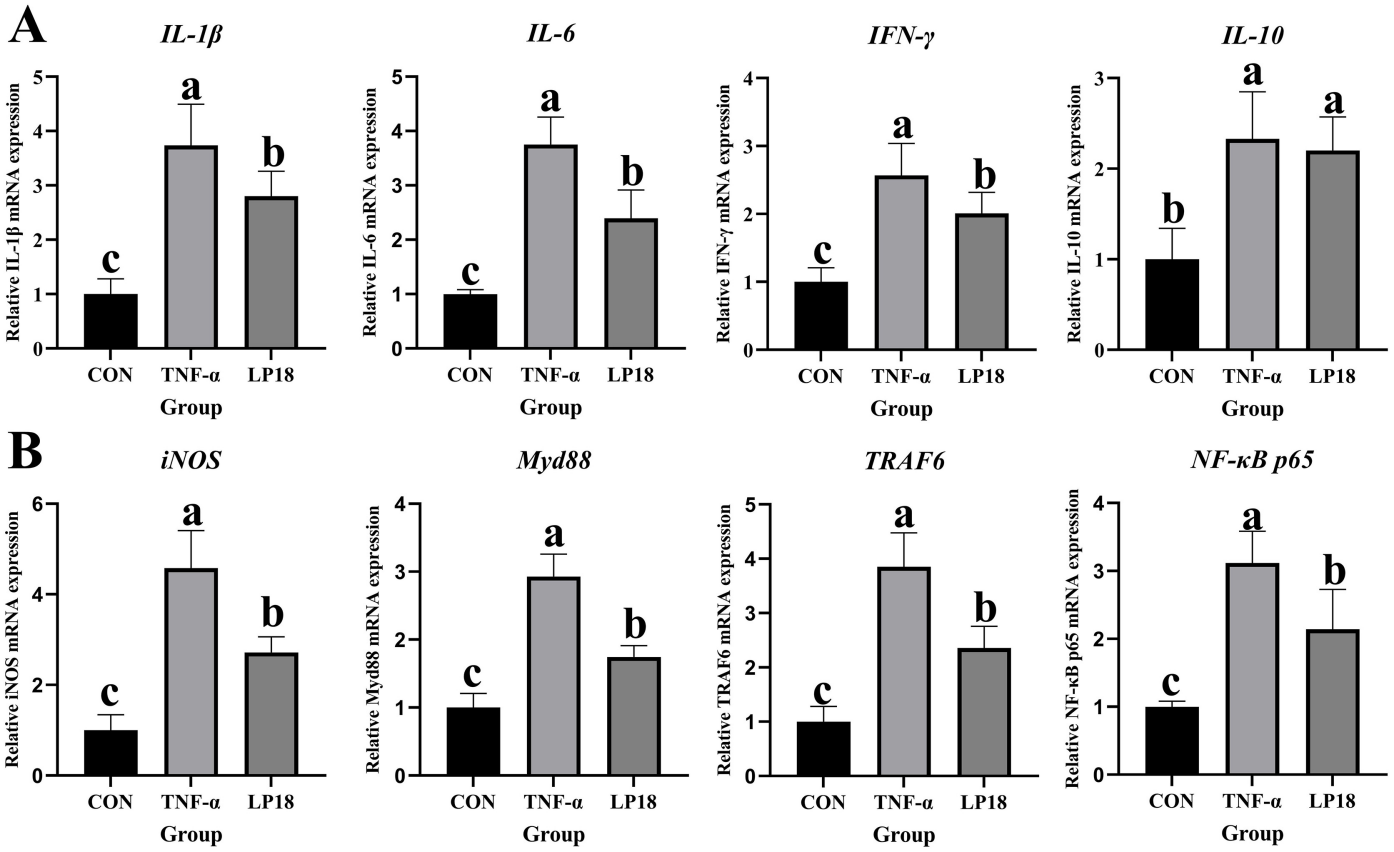
Expression of inflammatory cytokines and MyD88-NF-κB pathway related genes in CON, TNF-α, and LP18 groups. **(A)** mRNA expression of *IL-1β*, *IL-6, IFN-γ*, and *IL-10*. **(B)** mRNA expression of *iNOS, MyD88, TRAF6*, and *NF-*κ*B p65*. Data represent mean ± SD (*n* = 4). Distinct superscript letters ^(a,b,c)^ denote statistically significant intergroup differences (*P* < 0.05).

Pro-inflammatory cytokine expression was assessed in TNF-α treated epithelial cells (Figure 11A). TNF-α markedly increased the mRNA levels of *IL-1*β, *IL-6*, and *IFN-*γ compared with the control group. LP18 supplementation significantly reduced these cytokines relative to TNF-α treatment, although values remained above baseline. IL-10 expression was elevated in TNF-α treated cells and remained similarly high in the LP18 group.

To further examine inflammatory signaling, mRNA expression of *iNOS, MyD88, TRAF6*, and *NF-*κ*B p65* was measured (Figure 11B). All four markers were significantly upregulated in response to TNF-α stimulation. LP18 treatment reduced the expression of *iNOS, MyD88, TRAF6*, and *NF-*κ*B p65* compared with TNF-α, indicating partial suppression of TNF-α induced inflammatory pathway activation.

## 4 Discussions

Severe acute pancreatitis (SAP) is distinguished by widespread pancreatic inflammation, and a high incidence of intestinal barrier dysfunction, which worsens disease severity by facilitating bacterial translocation and endotoxin absorption (Chen J. W. et al., 2024). Although the gut-pancreas axis has received increased interest in SAP, the role of gut microbial metabolites and downstream inflammatory signaling pathways in intestinal damage is still unclear. Our study demonstrated that LP18 dramatically reduced SAP-induced intestinal inflammation and epithelial barrier breakdown. LP18 has protective benefits via restoring gut microbiota composition, increasing microbial butyrate synthesis, and inhibiting NF-κB pathway activation. It should be noted that LP18 was administered for 28 days prior to SAP induction, and therefore the present study is based on a preventive experimental design rather than a therapeutic intervention model. Accordingly, the protective effects observed herein primarily reflect the ability of LP18 to precondition the intestinal environment, enhance epithelial barrier integrity, and modulate microbial and inflammatory homeostasis before the onset of acute pancreatic injury. Our results point to a microbiota-metabolite-inflammation nexus that underpins LP18's therapeutic potential in SAP.

SAP-induced intestinal injury is characterized by mucosal shrinkage, epithelial apoptosis, and loss of barrier integrity (Chen J. W. et al., 2024). Consistent with prior studies, SAP animals' model in this work showed significant villus shortening, epithelial architectural breakdown, and downregulation of tight junction proteins (Dourado et al., 2018; Cui et al., 2019). The jejunum represents a major site of nutrient absorption and epithelial turnover and is highly sensitive to systemic inflammatory and metabolic stress in the experimental context studied. We found that LP18 therapy reduced these pathogenic alterations in jejunum, indicating that it promotes epithelial shape and barrier function during severe systemic inflammation. One key discovery is that LP18 has a strong anti-inflammatory impact. Excessive production of pro-inflammatory cytokines such TNF-α, IL-1β, and IL-6 by SAP might negatively impact intestinal epithelial function (Wang X. H. et al., 2022; Zhao et al., 2025). LP18 dramatically decreased the production of these cytokines, which is consistent with the immunomodulatory properties previously reported for several *Lactobacillus* strains. Notably, the anti-inflammatory impact seems to be mediated indirectly, via microbial and metabolic repair, rather than directly by immune suppression. This divergence was important because it implies that LP18 does not just suppress host immune responses, but rather modifies upstream microbial causes of inflammation. Furthermore, LP18 supplementation restored the SAP-induced tight junction disruption.

Increasing data suggests that gut microbiota dysbiosis is an active cause of illness aggravation rather as a subsequent outcome of SAP (Huang et al., 2024; Mao et al., 2025). SAP affects microbial ecology, degrades metabolic pathways, and weakens host mucosal defense, resulting in a pathogenic feedback loop that exacerbates intestinal barrier damage and leads to endotoxemia (Jin et al., 2022). Our findings are consistent with prior research, indicating that SAP induces a fast reduction in microbial diversity and profound structural alterations in the gut microbiota. SAP mice in our model showed lower microbial diversity and a significant drop in beneficial taxa such as *Faecalibacterium, Roseburia, Akkermansia*, and other SCFA-producing genera, which is consistent with prior research (Zou et al., 2025). These taxa have critical roles in mucosal homeostasis and anti-inflammatory signaling (Mei et al., 2021). SAP also encouraged the growth of opportunistic infections and facultative anaerobes, which may further compromise epithelial function and produce toxic metabolites (Wang et al., 2022a). LP18 administration improved microbial community balance by restoring α-diversity and reversed the loss of beneficial commensals. This conclusion is significant since *Lactobacillus* strains have long been regarded as probiotics, but emerging data suggests that their benefits are extremely strain-specific and disease-context dependent. Our results demonstrated LP18 increased the abundance of traditional butyrate-producing species such *Roseburia, Faecalibacterium*, and *Eubacterium*. These taxa are essential for sustaining epithelial energy supply, controlling mucosal immunity, and generating SCFAs via the acetyl-CoA and butyryl-CoA pathways. Their recovery by LP18 shows that this strain improves metabolic cross-feeding networks in the microbial community. Unlike strains that may increase dysbiosis in inflammatory situations, LP18 had a corrective impact, moving the microbial ecology toward a more protective makeup.

Given the critical involvement of the gut-pancreas axis in SAP, efforts for restoring intestinal homeostasis, notably via microbiota and metabolite manipulation, have sparked significant scientific interest (Glaubitz et al., 2023). Our integrated metabolomics investigation demonstrated that *Lactobacillus paracasei* LP18 significantly alters the gut metabolic landscapes altered by severe acute pancreatitis (SAP). Untargeted metabolomics revealed that SAP causes a wide metabolic collapse, defined by lower levels of important microbial-derived metabolites, altered glucose fermentation pathways, and disrupted amino acid metabolic networks. Principal component analysis strongly distinguished the SAP group from the healthy controls, demonstrating a worldwide metabolic change linked to intestinal damage and dysbiosis. Surprisingly, LP18 therapy partly corrected this metabolic disturbance, restoring several metabolite clusters linked to short-chain fatty acid (SCFA) production, glycolysis-derived intermediates, and energy metabolism.

Short-chain fatty acids (SCFAs) are crucial for intestinal health, with butyrate being particularly important for epithelial integrity (Ammer-Herrmenau and Neesse, 2024). Butyrate is the primary energy source for colonocytes, enhances tight junction expression, stimulates mucin production, and has strong anti-inflammatory and antioxidant properties (Li H. T. et al., 2024; Lv et al., 2025). Targeted SCFA metabolomics further demonstrated that LP18 has a substantial regulatory influence on butyrate metabolism. SAP mice showed a considerable decrease in luminal butyrate content, which is compatible with the depletion of traditional butyrate-producing genera and the collapse of microbial cross-feeding networks (Ye et al., 2021; Wang et al., 2022a). LP18 treatment dramatically raised butyrate levels, as well as critical metabolic precursors such as acetate and lactate, which aid in butyrate production via cooperative microbial processes. These data indicate that LP18 enhances the recovery of SCFA metabolic flow, most likely by increasing substrate availability, promoting ecological stability among fermentative bacteria, and facilitating the reassembly of butyrate-producing microbial consortia. Functionally, LP18-induced butyrate synthesis was strongly related with improvements in intestinal barrier integrity. Histological analysis showed that LP18 significantly decreased villus atrophy, epithelial shedding, and mucosal erosion in SAP mice. At the molecular level, LP18 restored tight junction protein expression, decreased epithelial apoptosis, and inhibited inflammatory signaling pathways including NF-κB and IL-1β. These protective effects are consistent with butyrate's well-known roles in promoting epithelial energy supply, increasing mucin secretion, stimulating epithelial regeneration, and inhibiting pro-inflammatory cytokine production. Although LP18 supplementation was associated with a significant increase in butyrate levels, the precise mechanisms underlying this metabolic shift warrant careful interpretation. Notably, the present study did not directly examine microbial functional gene expression or enzymatic activity related to SCFA biosynthesis. Therefore, it cannot be concluded that LP18 enhances butyrate production through direct transcriptional activation of butyrate-synthetic pathways. Instead, the observed elevation of butyrate is more likely attributable to LP18-induced restructuring of the gut microbial ecosystem, including enrichment of endogenous SCFA-producing taxa and/or promotion of metabolic cross-feeding interactions within the microbial community. Importantly, *Lactobacillus paracasei* is not considered a major primary butyrate producer; rather, it may function as a keystone modulator that indirectly supports butyrate production by shaping microbial composition and metabolic niches.

NF-κB activation is a key modulator of SAP-induced intestinal inflammation (Li X. et al., 2024). Excessive NF-κB activation triggers the production of pro-inflammatory cytokines, including TNF-α, IL-1β, and IL-6, which may cause epithelial apoptosis, tight junction disruption, and barrier failure (Zhang and Xu, 2023; Liu et al., 2025). Our investigation found that LP18 efficiently decreased NF-κB activation in SAP mice, leading to reduced cytokine production and better epithelial architecture. Restoring butyrate levels may be an upstream method for inhibiting NF-κB. Butyrate inhibits histone deacetylase (HDAC), promoting anti-inflammatory gene expression and inhibiting NF-κB nuclear translocation (Peng et al., 2024). Additionally, butyrate enhances peroxisome proliferator-activated receptor-γ (PPAR-γ) activity, which further antagonizes NF-κB signaling (Lu et al., 2024; Zhang et al., 2025). The observed decrease in NF-κB activation after LP18 administration might be due to epigenetic regulation, receptor-mediated signaling, and metabolic reprogramming. Correlation studies that included microbiota composition, global metabolomics, and SCFA profiling revealed robust positive connections between butyrate levels and epithelial barrier indicators, while inflammatory cytokines had negative correlations. These findings suggest a causal relationship between LP18-mediated metabolic restoration and enhanced intestinal barrier function. Overall, these findings suggest that LP18 improves SAP-induced intestinal barrier dysfunction not only by reshaping microbial composition, but also by restoring microbial metabolic activity, particularly butyrate production, via coordinated regulation of non-targeted metabolic pathways and SCFA-specific biosynthetic routes. Enhancement of butyrate production seems to be a key mechanism by which LP18 provides mucosal protection and restores gut homeostasis during SAP. Consistent with this interpretation, the correlation between increased butyrate levels and improved intestinal barrier integrity, along with suppressed NF-κB activation, supports a contributory role of butyrate in LP18-mediated protection. However, in the absence of direct functional manipulation, such as exogenous butyrate supplementation, inhibition of butyrate receptors, or microbial gene-level analyses—the current findings should be interpreted as associative rather than causative. Future studies integrating metagenomic or metatranscriptomic profiling and targeted functional interventions will be necessary to definitively establish whether SCFA biosynthesis represents a primary mechanistic axis of LP18 action. The present findings suggest that the protective effects of *Lactobacillus paracasei* LP18 are primarily mediated through local actions within the gut, rather than through direct systemic or organ-specific mechanisms. LP18 supplementation markedly improved intestinal barrier integrity, reshaped gut microbial composition, enhanced microbial metabolite profiles, and suppressed mucosal inflammatory signaling. These local intestinal effects likely play a central role in limiting luminal antigen translocation and dampening downstream inflammatory amplification.

## 5 Conclusion

In conclusion, *Lactobacillus paracasei* LP18 attenuates SAP-associated intestinal inflammation and barrier dysfunction, primarily through modulation of gut microbial composition, restoration of butyrate-associated metabolic profiles, and suppression of NF-κB–related inflammatory signaling. These findings support a gut-centered, microbiota-mediated protective mechanism and highlight the importance of microbial metabolic homeostasis in maintaining intestinal barrier integrity during severe inflammatory conditions.

## Data Availability

The original contributions presented in the study are publicly available. This data can be found here: https://www.ncbi.nlm.nih.gov/, accession number PRJNA1415544.
