# Supplementary figures and images for: Lactobacillus paracasei LP18 ameliorated inflammation and intestinal barrier dysfunction in severe acute pancreatitis via gut microbiota-mediated regulation of butyrate metabolism
Source: Front Microbiol. 2026 Feb 12;17:1765127. doi: 10.3389/fmicb.2026.1765127 (PMC12936515; doi:10.3389/fmicb.2026.1765127)

## Claudin-1

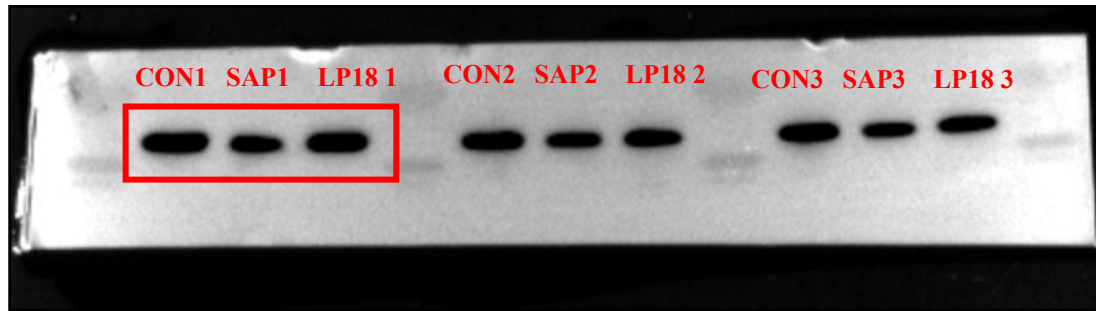

## Occludin

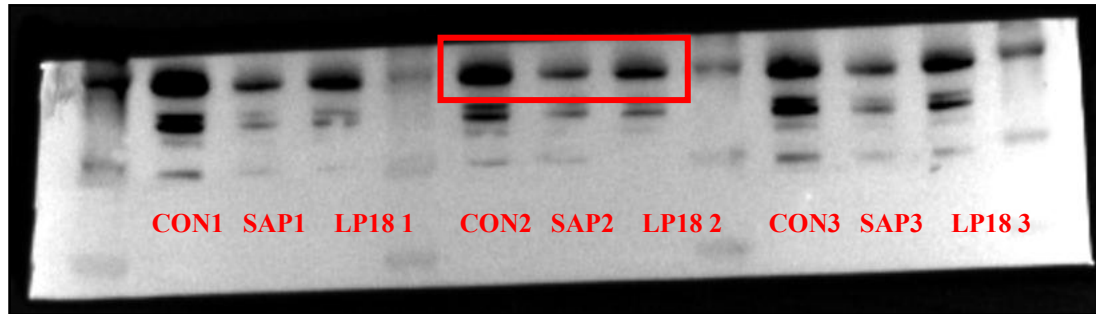

## $\beta$ -actin

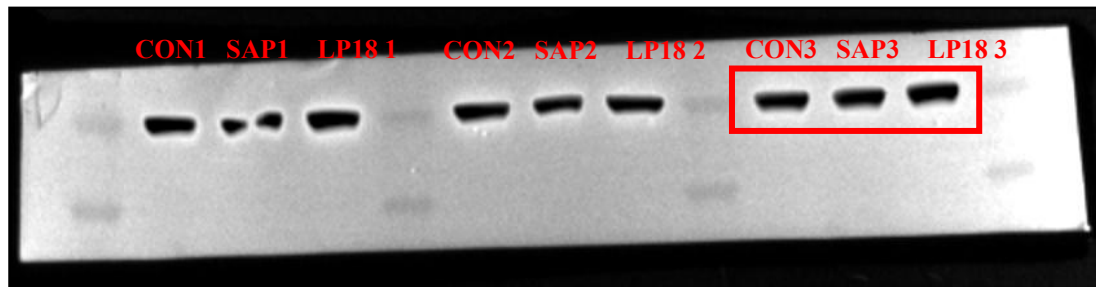

**TRAF6**

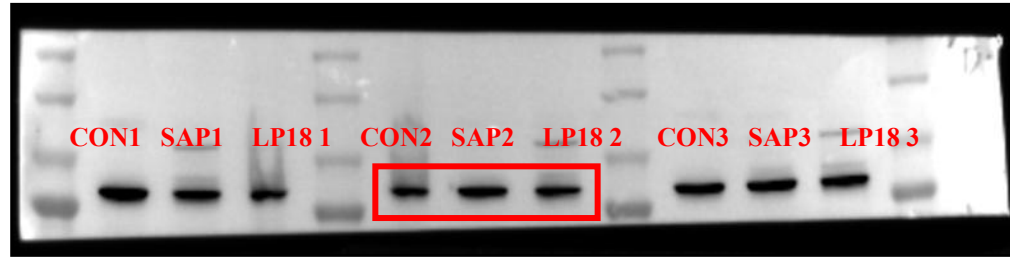

**$\beta$ -actin**

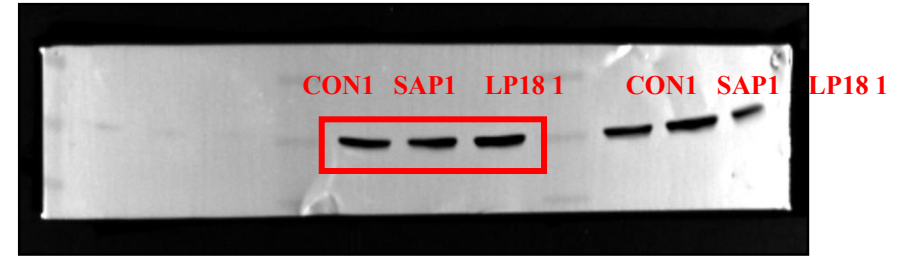

**NF- $\kappa$ B p65**

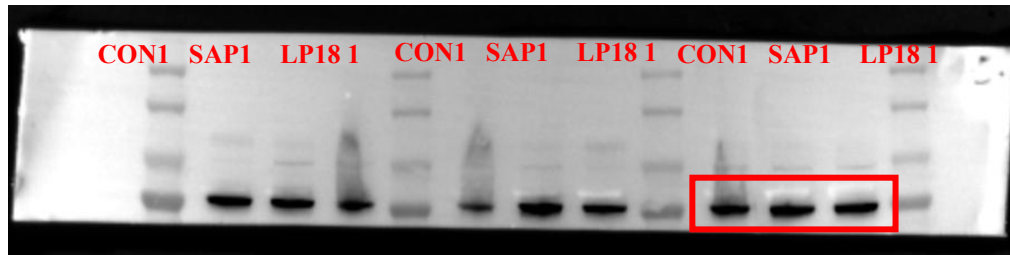

**p NF- $\kappa$ B p65**

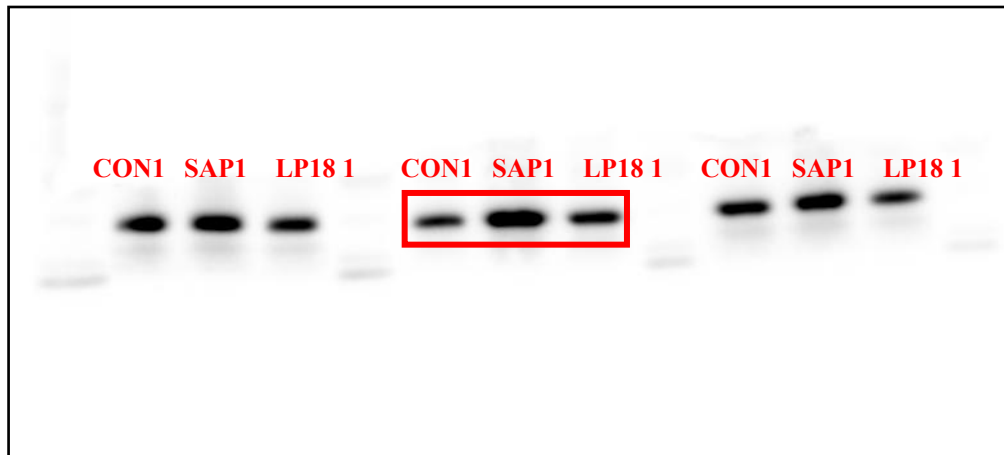

Supplement: Supplementary file 1 [file Image_1.pdf]
