# Supplementary material for: Lactobacillus paracasei LP18 ameliorated inflammation and intestinal barrier dysfunction in severe acute pancreatitis via gut microbiota-mediated regulation of butyrate metabolism
Source: Front Microbiol. 2026 Feb 12;17:1765127. doi: 10.3389/fmicb.2026.1765127 (PMC12936515; doi:10.3389/fmicb.2026.1765127)
Supplement: Supplementary file 2 [file Table_1.docx]

**Table S1 Composition of qRT-PCR system**

| **Reaction system composition** | **Dosage** |
| --- | --- |
| 2 × ChamQ Universal SYBR Qpcr Master Mix | 10 µL |
| PCR Forward Primer | 0.4 µL |
| PCR Reverse Primer | 0.4 µL |
| d H_2_O (Sterilize distilled water) | 7.2 µL |
| cDNA template 200 ng/µL | 2 µL |
| Total | 20 µL |

**Table S2 Program settings of qRT-PCR**

| Item | Program | cycle | temperature | time |
| --- | --- | --- | --- | --- |
| Stage1 | Pre-denaturation | 1 | 95℃ | 30 seconds |
| Stage2 | Circular reaction | 40 | 95℃ | 10 seconds |
|  |  |  | 60℃ | 30 seconds |
| Stage3 | Melting curve | 2 | 95℃ | 15 seconds |
|  |  |  | 60℃ | 60 seconds |
|  |  |  | 95℃ | 15 seconds |

**Table S3. Sequences of the oligonucleotide primers used for quantitative real-time PCR^1^.**

| **Gene Name** | **Forward Sequence (5’→3)** | **Reverse Sequence (5’→3)** | **Accession Number** |
| --- | --- | --- | --- |
| *β-actin* | TATAAAACCCGGCGGCGCA | TCATCCATGGCGAACTGGTG | NM_007393.5 |
| Claudin-1 | CAACCCGAGCCTTGATGGTA | AGATAAGCGAACCTGCCTCG | NM_016674.4 |
| Claudin-5 | GAAGGCATCTAGAAAACGACCAG | GAAGGCATCTAGAAAACGACCAG | NM_013805.4 |
| ZO-1 | GAAGTTACGTGCGGGAGCA | CCGTCCGCATAAACATCTCC | NM_001163574.2 |
| Occludin | GTCCTCCTGGCTCAGTTGAA | AGATAAGCGAACCTGCCTCG | NM_001360536.1 |
| GPR41 | GGATTACCCAGGGCTGGAAC | GAAAACGCTCACAAGGGCAG | NM_001033316.2 |
| GPR43 | GCACTGGACCAGAGGAGAAC | TTGGGCAAGTTCAGGGGTTT | NM_001168509.1 |
| GPR109A | GTTCTCCAAGTCTCCAAAGGTGGT | ACTAAGAAACGCAGACAGAGGAG | NM_030701.3 |
| IL-1β | TGCCACCTTTTGACAGTGATG | CAAAGGTTTGGAAGCAGCCC | NM_008361.4 |
| IL-6 | AGCCAGAGTCCTTCAGAGAGAT | TGACTCCAGCTTATCTCTTGGTT | NM_001314054.1 |
| IFN-γ | GTGATTGCGGGGTTGTATCTG | ACTGCAGCTCTGAATGTTTCTT | NM_008337.4 |
| IL-10 | TAAGGCTGGCCACACTTGAG | AGTAGGGGAACCCTCTGAGC | NM_010548.2 |
| iNOS | CTGGGAGCGCTCTAGTGAAG | CTCTCCACTGCCCCAGTTTT | NM_001313921.1 |
| Myd88 | ACTGATGCGGAGCCAGATTC | TGGGAGGAAAGGCAGTCCTA | NM_010851.3 |
| TRAF6 | ATATGACAGCCACCTCCCCT | TTGGCGTCCATGACCTCTTC | NM_001303273.1 |
| NF-κB p65 | TGCCTGGCCAGTGTAGCAGTCTT | CAAAGTCACCAAGTGCTCCACGAT | NM_010907.2 |
